# Mammary epithelial cells isolated from milk are a valuable, non-invasive source of mammary transcripts

**DOI:** 10.3389/fgene.2015.00323

**Published:** 2015-10-28

**Authors:** Marion Boutinaud, Lucile Herve, Vanessa Lollivier

**Affiliations:** ^1^UMR 1348 PEGASE, Institut National de la Recherche AgronomiqueSaint Gilles, France; ^2^UMR 1348 PEGASE, AGROCAMPUS OUESTRennes, France; ^3^Université Européenne de BretagneRennes, France

**Keywords:** lactation, mammary gland, ruminant, RNA, milk mammary epithelial cells

## Abstract

Milk is produced in the udder by mammary epithelial cells (MEC). Milk contains MEC, which are gradually exfoliated from the epithelium during lactation. Isolation of MEC from milk using immunomagnetic separation may be a useful non-invasive method to investigate transcriptional regulations in ruminants’ udder. This review aims to describe the process of isolating MEC from milk, to provide an overview on the studies that use this method to analyze gene expression by qRT PCR and to evaluate the validity of this method by analyzing and comparing the results between studies. In several goat and cow studies, consistent reductions in alpha-lactalbumin mRNA levels during once-daily milking (ODM) and in *SLC2A1* mRNA level during feed restriction are observed. The effect of ODM on alpha-lactalbumin mRNA level was similarly observed in milk isolated MEC and mammary biopsy. Moreover, we and others showed decreasing alpha-lactalbumin and increasing *BAX* mRNA levels with advanced stages of lactation in dairy cows and buffalo. The relevance of using the milk-isolated MEC method to analyze mammary gene expression is proven, as the transcript variations were also consistent with milk yield and composition variations under the effect of different factors such as prolactin inhibition or photoperiod. However, the RNA from milk-isolated MEC is particularly sensitive to degradation. This could explain the differences obtained between milk-isolated MEC and mammary biopsy in two studies where gene expression was compared using qRT-PCR or RNA Sequencing analyses. As a conclusion, when the RNA quality is conserved, MEC isolated from milk are a valuable, non-invasive source of mammary mRNA to study various factors that impact milk yield and composition (ODM, feeding level, endocrine status, photoperiod modulation, and stage of lactation).

## Introduction

In the mammary tissue, transcriptomic regulations drive the process of lactation (Bionaz et al., 2012). Controlling the expression of genes involved in milk synthesis, cell turnover, or hormone response in the mammary tissue is determinant for milk production in ruminants. Moreover, the immune response to mammary infections such as mastitis has also been shown to depend on transcriptional regulations in the mammary tissue (Whelehan et al., 2011). However, studying transcriptomic regulations responsible for changes in milk yield and composition or in the immune response entails the collection of mammary epithelial cells (MEC). Thus, MEC must be harvested from the mammary gland. The classic method is collection of mammary tissue by biopsy. However, this requires the performance of a surgical procedure (local anesthesia, skin incision with a scalpel, extraction of mammary tissue using a rotating blade, pressure to the udder to control hemorrhage and closure of the wound with a skin stapler). This procedure does not allow easy and repetitive sampling without damaging the mammary tissue. From an ethical point of view, but also based on scientific considerations, it is important to find alternative procedures to surgical ones whenever possible, also avoiding the carry-over effect of sampling that may be observed in mammary biopsies. Previous studies show that milk mostly contains immune system cells (lymphocyte, macrophage, neutrophils), but also viable MEC (Boutinaud and Jammes, 2002). The development of an immunomagnetic method enables the isolation of MEC from human milk somatic cells (Alcorn et al., 2002). This method has been adapted to ruminant milk (Boutinaud et al., 2008). This review aims to demonstrate that milk-purified MEC cells can be a valuable, non-invasive source of mammary transcripts.

## The Methodology for Using MEC from Milk to Analyze Mammary Gene Expression

### The Process of MEC Isolation from Milk

Milk contains several cell types; most of which are immune system cells, and a minority of MEC. The first step of MEC isolation from milk for RNA studies is low-speed centrifugation (<2,800 × *g*) in a conical flask to pellet the cells at the bottom of the flask. Since MEC concentration in milk is low, sufficient volumes of milk must be centrifuged in order to obtain enough RNA for gene expression analyses. Goat milk contains a higher concentration of MEC than bovine milk (Boutinaud and Jammes, 2002). Thus, 1.8–3.6 kg of bovine milk (Boutinaud et al., 2008, 2012, 2014; Krappmann et al., 2012; Sigl et al., 2014), 0.9–1.6 kg of caprine milk (Ben Chedly et al., 2011, 2013) and 1 kg of buffalo milk (Yadav et al., 2014) were used to extract purified MEC from milk in order to analyze mammary transcripts. During the second step of MEC isolation, an immunomagnetic separation technique is used to isolate the MEC from the total milk somatic cells and to remove the leukocytes. After several washings, total milk cell suspension is either directly incubated with magnetic beads coated with a specific antibody (Boutinaud et al., 2008) or indirectly, first incubated with the antibody and then with the magnetic beads (Sigl et al., 2012). After incubation on a rotary mixer at 4°C, the antibody-bound cells are then collected by placing the vials into a magnetic particle concentrator, thus the immune cells are discarded. After this step, the cell viability of milk-purified MEC suspensions were assessed using trypan blue staining showing that the majority of milk MEC are viable with an average of 62–77% and 83% cell viability in twice daily milked dairy cows (Boutinaud et al., 2012; Lollivier et al., 2015) and goats (Ben Chedly et al., 2013), respectively. The last step is centrifugation, used to pellet the purified MEC, followed by RNA extraction performed using various methods.

### Checking the RNA Quality is Crucial before Using MEC from Milk for Transcript Analysis

Mammary epithelial cells from milk are shed from the mammary epithelium. As such, they are no longer in contact with the extracellular matrix known to provide survival signals to MEC in the mammary tissue (Katz and Streuli, 2007). Moreover, they are not connected to each other by tight junctions. In addition, the induction of tight junction disruption is known to cause gene expression changes and cell apoptosis in mammary tissue (Ben Chedly et al., 2010). Even though MEC from milk are disconnected from their original environment, they are not completely dead, as they can still be cultivated (Ben Chedly et al., 2010; Sorg et al., 2012). Outside of their natural environment, milk-purified MEC can be fragile. Moreover, milk contains ribonuclease (Dalaly et al., 1980), thus their RNA may be susceptible to degradation. Despite all this, in most studies, the RNA quality of milk-purified MEC is acceptable for gene expression analyses as assessed using the RNA Integrity Number (RIN) generated by Agilent 2100 expert software (Schroeder et al., 2006) with RIN above 8. However, poor RNA quality in milk-purified MEC could partially explain the absence of concordance between gene expression in the mammary tissue and in milk-purified MEC in two studies. In a study where cows were fed with a diet rich in plant oil and docosahexaenoic acid-rich algae (DHA)-, the RNA quality of milk-purified MEC samples did not reach full satisfactory quality (Angulo et al., 2012). The lipid and DHA-rich algae supplementations resulted in a tendency to reduce the RNA quality (RIN < 7) in milk-purified MEC. In this study, there was a joint down-regulation of mammary lipogenic enzyme gene expression (stearoyl-CoA desaturase, *SCD*1, FA synthase, *FASN*, and sterol regulatory element binding transcription factor 1, *SREBF1*) in the mammary tissue, and a lack of effect in milk-purified MEC. Similarly in another study, gene expression in milk-purified MEC was compared to total milk somatic cells, biopsy, laser-microdissected MEC and milk fat globules using RNA Sequencing (Cánovas et al., 2014). Unfortunately in that study, the RNA quality of milk-purified samples (*n* = 3) was not optimal (RIN = 6) with a large proportion of low molecular weight RNA. This could be due to the specificity of this study with a milk storing time of 3-h before performing MEC purification. The RNA Sequencing analysis showed a high correlation of gene expression between milk somatic cells, mammary biopsy, laser-microdissected MEC and milk fat globule samples, while peculiarities were observed with milk-purified MEC, such as surprising, relatively low levels of β-lactoglobulin (*BLG)*, α-lactalbumin (*LALBA)* and *GLYCAM-*1 (Cánovas et al., 2014). The particularly poor RNA quality of these milk-purified MEC samples may partly explain the discrepancies with the other sources of mammary RNA. These two studies suggest that RNA from milk-isolated MEC is sensitive to degradation. Since, according to Fleige and Pfaﬄ (2006), the analysis of RNA levels is greatly influenced by the RNA’s integrity, we suggest that it is crucial to consider the RNA quality before using these cells as a source of mammary transcripts.

### The Importance of MEC Purification in Quantifying Mammary Gene Expression

The importance of using a purification steps to collect MEC is questionable. Indeed, compared with total milk somatic cells as a source of mammary transcripts, less RNA is obtained using milk-purified MEC. The small amount of RNA recovery is not a problem using qRT-PCR, but requires amplification before transcriptomic analysis, such as RNA Sequencing analyses and an amplification step can introduce bias across samples with overexpression of some genes (Vallandingham et al., 2013). Moreover, purification is a time consuming step, as such it can have a negative influence on RNA quality. Thus, avoiding this step, while using total milk somatic cells, would be better to preserve RNA quality. However, purification does not always have the same impact on RNA quality depending on the studies. In contrast with a recent study (Cánovas et al., 2014), the quality of RNA was better in milk-purified MEC than in total milk somatic cells (RIN 8.0 vs. 4.1, *P* < 0.05; Boutinaud et al., 2008).

The use of the purification method also provides advantages over the use of somatic cells for the quantification of gene expression. Firstly, the use of total milk somatic cells as a source of mammary RNA may be arguable for some genes of interest that are not solely expressed in MEC and also expressed in leukocytes. Likewise for genes involved in apoptosis pathways, most of them are not solely specific of MEC. It is for example the case for *BAX* which is expressed in all types of cells, especially in leukocytes, known to undergo spontaneous apoptosis (Paape et al., 2000). Although, more and more studies reported the use of milk somatic cells to analyze the expression of genes involved in milk synthesis in cows (Murrieta et al., 2006; Feng et al., 2007; Wickramasinghe et al., 2012), goats (Tudisco et al., 2014), sheep (Mura et al., 2013), and yaks (Bai et al., 2013) using northern blot, RT PCR analyses, or RNA sequencing, milk somatic cells were not considered as suitable for measuring milk protein expression in lactating ruminant (Sciascia et al., 2012). The use of total milk somatic cells may also be problematic with the real time RT-PCR technique for analyzing gene expression due to the need for a suitable reference gene (Dheda et al., 2005). Total milk cells are a mixed cell population and therefore subjected to change in subpopulation fractions. As an example the proportion of epithelial cells among total somatic cells varies from one sample to another (Boutinaud and Jammes, 2002). The changes in the subpopulation fraction potentially affect the choice of the most suitable reference gene. Accordingly, several studies reported the necessity of finding a suitable reference gene for studying gene expression in milk somatic cells in various species such as goats (Modesto et al., 2013; Jarczak et al., 2014), zebu (Varshney et al., 2012), or yaks (Bai et al., 2014). The variation in the subpopulation fraction can also be a problem when using a technique such as RNA Sequencing as it takes into account all the genes expressed in a given sample. If the proportion of MEC varies, it will impact the number of genes expressed. Even in milk-purified MEC, the reference gene is a matter of debate. In one study, cytokeratin (*KRT18)* has been used as a reference gene (Krappmann et al., 2012). However, since no correlation of expression between udder- and milk-purified MEC samples was observed, the authors concluded that this method is not adequate to reflect metabolic processes. The use of KRT18 as the reference gene is not necessary when MEC are already purified. The choice of the reference gene must be evaluated based on the stability of the expression under the conditions of the experiment as previously carried out with milk-purified MEC (Yadav et al., 2012). However, the conventional use of a single gene for normalization leads to relatively large errors and the use of the geometric mean of multiple carefully selected housekeeping genes is necessary (Vandesompele et al., 2002). Thus, compared with using somatic cells as a source of mammary transcripts, the use of milk-purified MEC shows several advantages (analyzing non-epithelial specific gene and avoiding variations due to the proportion of MEC among milk somatic cells) for gene quantification using RT-PCR, but also RNA Sequencing analyses. Despite these arguments in favor to the use of a purification step, the results obtained in the RNAseq study showing a higher correlation of gene expression with mammary biopsy for milk somatic cells than for milk-purified MEC (Cánovas et al., 2014) discredits this method. The fact that quality of RNA of milk-purified MEC was not optimal is not sufficient to explain such differences. The low amount of RNA obtained with milk-purified MEC may have generated more bias after the amplification step used in that study. Nevertheless, other broadband transcriptomic analysis with higher number of samples must to be investigated in order to compare gene expression between milk somatic cell and milk-purified MEC and to clearly validate the use of milk-purified MEC as a source of mammary transcript.

### The Use of a Proper Antibody

**FIGURE 1:**
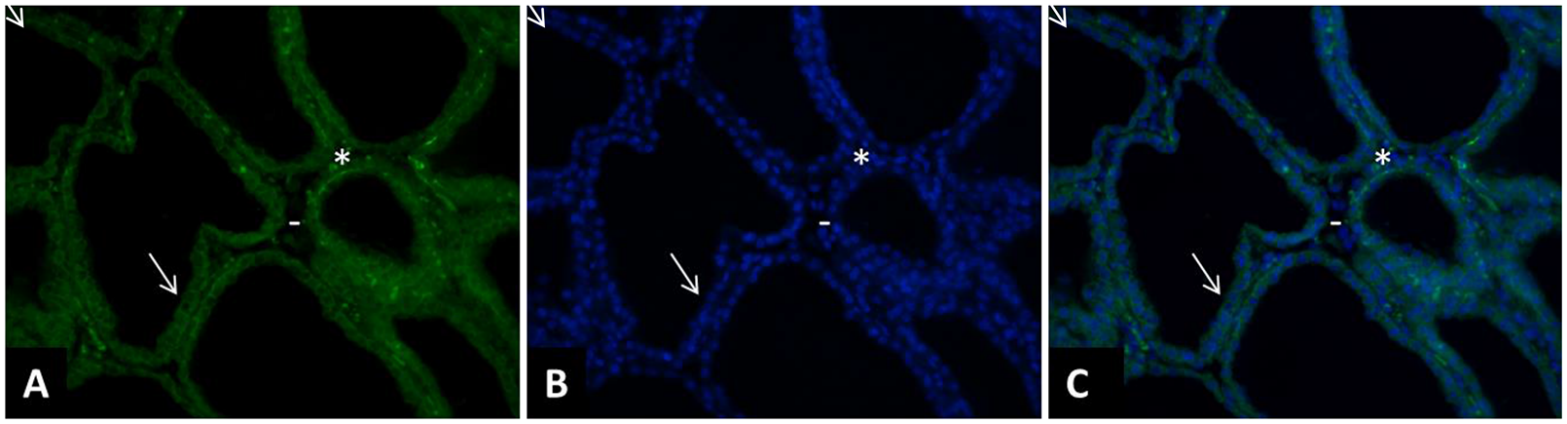
**Immunohistology of a cow mammary tissue during lactation (DIM = 77) stained with clone 34bE12 anti-cytokeratin antibody**. The mammary tissue sample was fixed in 4% paraformaldehyde for 2 h and paraffin-embedded. The tissue sections (5 μm thickness) were deparaffinized in three changes of a xylene bath and rehydrated in a graded ethanol–water bath series (100% ethanol, 90% ethanol, 70% ethanol, and distilled water). After rehydration, and several TBS washes, the tissue sections were permeabilized by 5 min microwave exposure in sodium citrate 10 mM. The tissue sections were pre-incubated for 10 min in TBS with 10% normal goal serum (NGS) and then incubated for 2 h with a monoclonal mouse –anti-cytokeratin antibody (clone 34bE12, 1:100, Dako, Trappes, France). After several TBS washes, the sections were incubated with an anti-mouse secondary antibody (1:400, Alexa Fluor 153 568 Goat anti-Mouse IgG, A11031, Invitrogen Life Technology, Berlin, Germany) for 30 min **(A,C)**. After being washed in TBS, the sections were incubated for 3 min with 4’,6-diamidino-2-phenylindole (DAPI; Sigma–Aldrich) at a concentration of 0.33 μg/mL **(B,C)**. All slides were mounted with VECTASHIELD (Valbiotech, Paris, France) and examined under fluorescence using a Nikon Eclipse E400 microscope (Nikon France, Le Pallet, France). The images were captured with a DXM 1200 digital still camera (Nikon France, magnification, 200×; area, 0.14 mm^2^ per microscopic field) were examined for each staining. The clone 34βE12 stained clearly luminal MEC (➘) but strong staining is also observed in the stroma part in some cells close to the luminal layer that may correspond to myoepithelial cells (^∗^). However, most of the cells in the stroma are not stained by the antibody (-).

In the first study reporting this technique, an antibody specific to cell surface antigen, epithelial membrane antigen (EMA), was used to purify MEC from human milk (Alcorn et al., 2002). In ruminants, in most studies the antibody used to purify MEC is directed against cytokeratin 8 which is specific to alveolar MEC (**Table 1**). Some more recent studies used the clone 34βE12, which was first shown to be reactive against keratin proteins 1, 5, 10, and 14. These cytokeratins are mostly expressed in myoepithelial cells, suggesting that this antibody is not appropriate to purify MEC from milk. However, this antibody strongly reacts with Lobular lesions in breast (Lobular Intraepithelial Neoplasia, (LIN; Bratthauer et al., 2002). And none of the individual clonal antibody directed against this individual cytokeratin (cytokeratin 1, 5, 10, and 14) reacted with the cells of LIN (Bratthauer et al., 2003). The antibody against cytokeratin 19 was the closest to demonstrating the reactivity seen with clone 34βE12 (Bratthauer et al., 2003). Cytokeratin 19 is well known to be specific of luminal MEC (Bartek et al., 1990). In non-neoplastic mammary tissue clone 34βE12 stains the cytoplasm of myoepithelial cells as well as luminal ductal epithelium (Dairkee et al., 1988; Esposito et al., 2007). Since most of the literature about clone 34βE12 concerns human mammary tissue and non-lactating tissue, we performed a staining with this antibody in bovine lactating mammary tissue and observed that the alveolar MEC are stained with the antibody (**Figure 1**). The clone 34βE12 stained clearly luminal MEC but strong staining is also observed in the stroma part in some cells close to the luminal layer that may correspond to myoepithelial cells. However, most of the cells in the stroma are not stained by the antibody. From the **Figure 1**, we can conclude that, together with anti-cytokeratin 8 antibodies, clone 34βE12 antibody is also appropriated for MEC purification.

**Table 1:** Antibody used to purify mammary epithelial cells from milk cell suspension using magnetic beads in ruminants.

Molecule	Clone	Manufacturer	Species	Reference
Anti-cytokeratin 8	Clone K8.13	Sigma–Aldrich Chimie, Lyon, France	Cow	Boutinaud et al., 2008
			Cow	Boutinaud et al., 2012
			Goat	Ben Chedly et al., 2011
			Goat	Ben Chedly et al., 2013
			Cow	Boutinaud et al., 2013a
			Buffalo	Yadav et al., 2012
			Buffalo	Yadav et al., 2014
Anti-cytokeratin 8	C5301	Sigma–Aldrich Chimie, Lyon, France	Cow	Wang et al., 2014
Anti-cytokeratin 8	Clone C-43	EXBIO, Prague, Czech Republic	Cow	Sigl et al., 2012
			Cow	Sigl et al., 2014
Anti-cytokeratin 1, 5, 10, and 14	Clone 34βE12	Dako, Trappes, France	Cow	Angulo et al., 2012
			Cow	Lollivier et al., 2015
			Cow	Boutinaud et al., 2013b
			Cow	Boutinaud et al., 2014
			Cow	Cánovas et al., 2014

### The Efficiency of MEC Purification

In order to verify the efficiency of the purification method, gene expression for several cytokeratins was analyzed in milk-purified MEC. Enriching MEC from whole milk somatic cells has been shown to be effective, as indicated by the higher levels of *KRT8* or *KRT18* mRNA in milk-purified MEC than in milk somatic cells (Boutinaud et al., 2008; Wang et al., 2014) and in mammary tissue (Krappmann et al., 2012). Moreover, milk-purified MEC samples were barely contaminated with immune cells, as they showed low mRNA abundance of specific leukocyte markers (Wang et al., 2014). In contrast in one bovine experiment, an over expression of CD68 in milk-purified MEC suggested a contamination with macrophages (Cánovas et al., 2014).

The reliability of the purification method can be evaluated in studies where different antibodies were used to specifically select the MEC (**Table 1**). The effect of feed restriction in dairy cows was analyzed in two studies using two different antibodies directed against cytokeratin 8 and in these both studies the effect of feed restriction was similar (Boutinaud et al., 2008; Sigl et al., 2014). Furthermore, the effect of Quinagolide, a prolactin-release inhibitor, was similar regardless of the anti-cytokeratin antibody used: anti-cytokeratin 8 (Boutinaud et al., 2012) or anti-cytokeratin 1, 5, 10, and 14 (Lollivier et al., 2015). In addition, the effect of the lactation stage was concordant in two bovine studies (Sigl et al., 2012; Boutinaud et al., 2013b) as well as one bovine study and one buffalo study (Boutinaud et al., 2013b; Yadav et al., 2014) where different antibodies were used (**Table 1**). Finally, the agreement of these different studies suggests that the immunomagnetic method of MEC purification is reliable.

## Factors Known to Govern Transcript Variation in Milk-Purified MEC

### The Effects of Feed Restriction

One of the initial factors of variation in mammary transcripts that has been studied using milk-purified MEC is the effect of feed restriction. In a first experiment, five Holstein dairy cows were submitted to an experimental design, which was a 2 × 2 factorial arrangement of two milking frequencies and two feeding levels. The experiment was divided into two main 3-week periods, during which cows were fed either 98 or 70% of their needs. Milk MEC were isolated after 2–3 weeks of feed restriction treatment. Short term feed restriction causes a decrease in milk yield (–13%) in comparison with the 98% feeding level treatment. This effect was accompanied by a decrease (–53%) in the transcript levels of *SLC2A1* (Boutinaud et al., 2008), one of the main transmembrane transporters of glucose in mammary gland (Zhao, 2014). This effect could in part explain the lower glucose uptake by the mammary gland during feed restriction. As a consequence of low glucose levels available for lactose synthesis, and since lactose is the major osmotic agent in milk, reduced lactose secretion may in part be responsible for the decrease in milk yield during feed restriction (Boutinaud et al., 2008).

The effect of short term feed restriction has been studied at different stages of lactation in Holstein–Friesian dairy cows (during early or mid-lactation). A reduction of *SLC2A1* mRNA level with feed restriction was observed in both lactation stages (Sigl et al., 2014). A similar reduction in *SLC2A1* with feed restriction is observed to that observed previously (Boutinaud et al., 2008). In contrast, the effects on milk protein mRNA levels and on a key regulator of milk protein synthesis were observed to be different according to the stage of lactation. Only in early lactation, the expressions of κ-casein (*CSN3*), *LALBA* and one transcription factor gene, E74-like factor 5 (ELF5), increased during short term feed restriction (Sigl et al., 2014). The absence of variation of these milk protein genes in a later stage of lactation is concordant with previously published results (Boutinaud et al., 2008). Thus, similar variations in mammary transcripts from milk-isolated MEC were observed under the effect of feed restriction in two bovine studies.

### The Effects of Once-daily Milking

One of the main factors of variation in mammary transcripts that has been studied using milk-purified MEC is the effect of once-daily milking (ODM) in comparison with a more common milking frequency, which is twice-daily milking (TDM). The practice of ODM resulted in a 7–50% decrease in milk yield depending on the species and breeds, but also on the duration and stage/timing of ODM (Marnet and Komara, 2008). Multiple studies investigated the effect of ODM on mammary transcripts using milk MEC collection in order to understand the cellular regulations involved in the reduction of milk yield during ODM.

The first studies were interested in analyzing the effect of ODM on several transcripts involved in milk protein and lactose synthesis. In the first experiment, milk MEC were collected from five cows submitted to a 2 × 2 factorial arrangement of two milking frequencies (ODM and TDM) and two feeding levels (98 or 70% of needs, Boutinaud et al., 2008). In cows that received the 98% feeding level, ODM decreased milk yield (–19%), milk protein (–17%), and milk lactose (–22%). These decreases during ODM were accompanied by reductions in *LALBA* and *CSN3* mRNA levels by –73 and –86%, respectively (**Table 2**). The effect of ODM on milk protein transcripts in milk-purified MEC was also studied in two others trials, where cows were unilaterally milked once-daily. In these two experiments, the loss of milk yield was high, with a reduction from –41% to –31% of milk produced in one udder half in comparison with the contralateral one. In both studies, a consistent reduction in *LALBA* mRNA levels with a similar range (on average –75%) was observed during ODM compared with TDM (**Table 2**).

**Table 2:** Effect of once-daily milking (ODM) compared to twice-daily milking (TDM) on milk yields and on two milk protein and *BAX* mRNA levels in milk-purified mammary epithelial cells (MEC) or mammary biopsy in cows and goats.

				Delta yield ODM/TDM					Delta mRNA level ODM/TDM			
Species	Treatment duration	*n*	Milk yield during TDM kg/d	Milk	Fat	Protein	Lactose	Lactose content	*LALBA*	*CSN3*	*BAX*	Reference
									**In milk purified MEC**			
Cow	1 week	10	12.4^1^	-19%^∗∗∗^	-13%^∗^	-17%^∗∗∗^	-22%^∗∗∗^	-2.3%^†^	-73%^∗∗^	-86%^∗∗∗^	762%^∗^	Boutinaud et al., 2008
Cow	1 week	4	19.1^1^	-41%^∗∗∗^	-33%^∗∗^	-38%^∗∗∗^	-46%^∗∗∗^	-	-76%^∗^	-61%^†^	NS	Boutinaud et al., 2012
Cow	1 week	5	17.6^1^	-31%^∗∗∗^	-25%^∗∗^	-30%^∗∗∗^	-32%^∗∗∗^	-4.2%^†^	-74%^∗^	-49%^†^	-	Boutinaud et al., 2014
Goat	5 weeks	8	3.1	-17%^∗∗∗^	-22%^∗∗∗^	-17%^∗∗^	-18%^∗∗∗^	NS	-88%^∗^	-40%^†^	189%^∗^	Ben Chedly et al., 2011
Goat	5 weeks	6	3.5	-23%^∗∗∗^	-20%^∗^	-16%^∗∗^	-23%	NS	-75%^∗^	-66%^†^	186%^∗^	Ben Chedly et al., 2013
Goat	3 weeks	10	2.5	-19%^∗∗∗^	-26%^∗∗∗^	-12%^∗^	-	-	-75%^∗∗^	-43%^∗^	-	Unpublished data
									**In mammary biopsies**			
Cow	1 week	5							-100%^†^	-98%^†^	-	Boutinaud et al., 2014
Goat	5 weeks	6							-29%^∗^	NS	NS	Ben Chedly et al., 2013
Goat	3 weeks	10							-27%^∗^	-27%^∗∗^	-	Unpublished data

*^∗∗∗^*P* < 0.001; ^∗∗^*P* < 0.01; ^∗^*P* < 0.05; and ^†^*P* < 0.10*.

*^1^Milk Yields in udder half*.

The effect of ODM was also studied using milk-purified MEC in three trials in goats. As for cows, ODM decreased milk yield and induced significant reductions in *LALBA* mRNA levels between –88% and –75%. For one of the goat experiments, the effect of ODM on *CSN3* mRNA levels was significant (–43%), whereas for the two others, only tendencies were observed. These results suggest that *LALBA* transcript has a key role in determining the milk yield during ODM. Indeed, *LALBA* gene, which encodes the coenzyme of lactose synthase, could help explain the lower milk production levels during ODM, given that lactose is a major osmotic agent.

In parallel with the analysis of the transcripts involved in milk protein and lactose synthesis, the effect of ODM on the expression of *BAX*, a gene coding for a pro-apoptotic factor was also investigated using milk-purified MEC. In most of the studies, one in cows and two in goats, ODM induced greater *BAX* RNA levels in milk-purified MEC (**Table 2**). We can hypothesize that the higher *BAX* mRNA level during ODM in milk-purified MEC could result of an induction of cell apoptosis in the mammary tissue after 24 h of milk accumulation in the mammary gland. A higher gene expression of *BAX* in milk MEC could also result from MEC remaining in the gland cistern for long periods, due to 24 h milk accumulation. However, our hypothesis is that most milk MEC cell are freshly exfoliated after myoepithelial cell contraction during milking. Further investigations on the characterization of MEC exfoliation in milk will give us more information about the use of *BAX* in milk-purified MEC as an indicator of the apoptotic process in the mammary tissue during ODM.

The effect of ODM using milk-purified MEC has been further studied, comparing its effect on mammary biopsy. The effects of ODM on mammary transcripts in milk-purified MEC were compared with those in mammary biopsies using real time RT-PCR analyses. In several goat and cow studies, we observed a consistent reduction in *LALBA* mRNA levels during ODM between milk-purified MEC and mammary biopsies (**Table 2**). Similarly to what is observed in milk-purified MEC, *CSN3* mRNA levels in the mammary tissue sampled by biopsy were not always significantly affected during ODM but followed a tendency for reduction in two studies out of three (*P* < 0.10, **Table 1**). In one of the cow studies, a larger panel of transcripts was compared between mammary biopsy and milk-purified MEC (Boutinaud et al., 2013a). In this study, the effect of ODM was analyzed in five Holstein cows subjected to unilateral ODM and TDM for 8 days. Similar down-regulations were observed in both milk-purified MEC and mammary tissue, regarding five transcripts, (*FABP3*, a fatty acid transporter; *ABCG2*, a carrier-associated secretion of xenobiotics; *SLC34A2*, a solute carrier and *RNASE1* and *RNASE5*, antimicrobial agents. Similarly in both mammary tissue and milk-purified MEC non-significant effect of ODM was observed for both *SCD* and *CSN3* transcripts. In addition, nucleobindin 2 (*NUCB2)* which is involved in cell proliferation and migration in human MEC (Suzuki et al., 2012), was similarly down-regulated in both mammary tissue and milk-purified MEC. However, the comparison of mRNA variations after ODM between milk-purified MEC and mammary biopsies also showed discrepancies. Three of the transcripts that were significantly down-regulated in mammary tissue (microarray analyses) were clearly not modified by ODM in milk-purified MEC (*PLIN2*, *CD36*, *and LPL*, *P* > 0.6). Although these RNA correspond to three proteins that are expressed in epithelial cells and have important function for milk fat synthesis (Bionaz and Loor, 2008), they are not epithelial cell-specific, but can also be expressed in other cells contained in mammary tissue. Given that *CD36* and *PLIN2* are involved in phagocytosis, their expressions in mammary tissue may be located in monocytes or macrophages, and are therefore not related to a reduction in milk fat synthesis, but rather to increased levels of apoptosis in this tissue, as a feedback effect after 8 days of unilateral ODM. Moreover, most of the transcripts upregulated in the mammary tissue involved in cell remodeling (cellular growth and proliferation, cell movement, and cell death) were stable in milk-purified MEC. For some transcripts, this can be explained by the fact that they belong to a category linked to non-epithelial mammary tissue, transcripts which – in theory – are not detected in milk-purified MEC and are weakly expressed in milk-purified MEC samples (such as *ITGB6*, the *IGFBP4* components of inflammatory system or the *COL1A1* component of connective tissue). However, IGFBP-5 which is known to be expressed in MEC (Boutinaud et al., 2004) was upregulated in mammary tissue and did not vary in milk-purified MEC during ODM. The absence of *IGFBP-5* up-regulation in milk-purified MEC during ODM is all the more surprising since *IGFBP5* is known to be involved in apoptosis in mammary gland (Boutinaud et al., 2004). The comparison between mammary tissue and milk-purified MEC must be further studied using broadband transcriptomic analysis.

**FIGURE 2:**
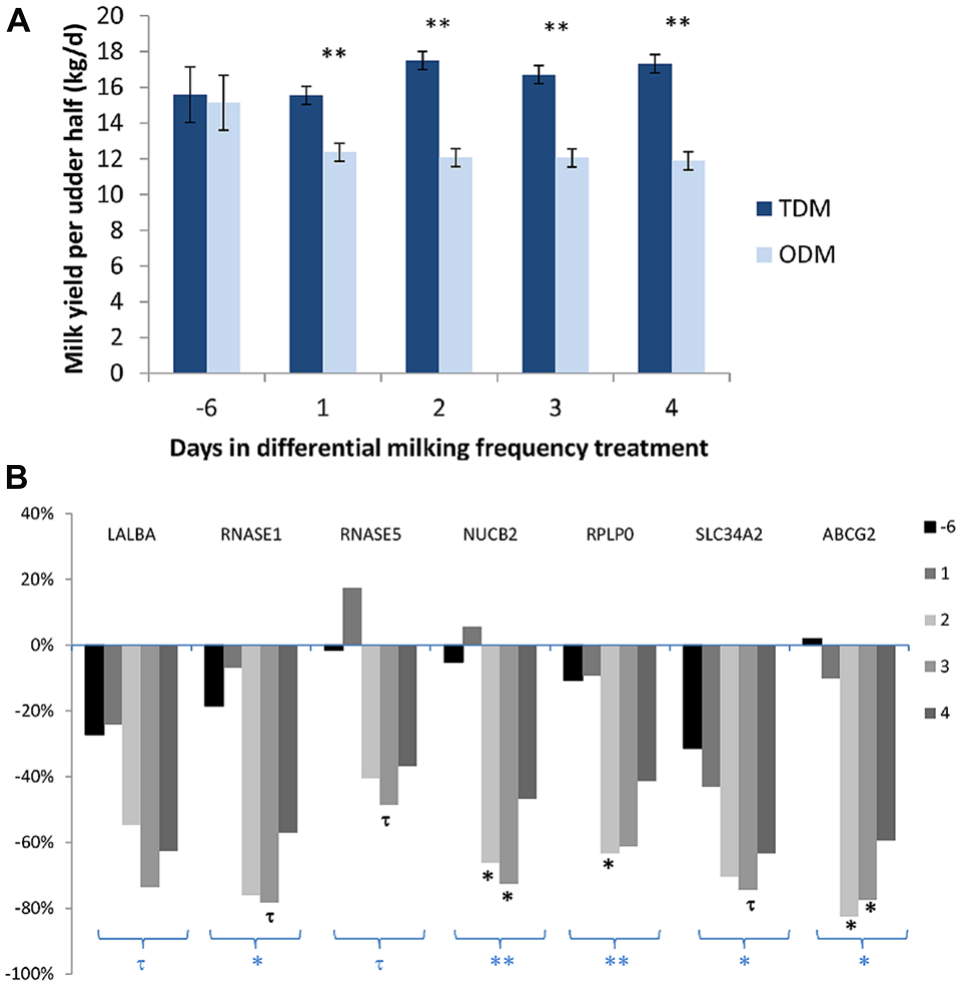
**Milk Yield (A) and variation in the mRNA levels of various genes (*LALBA, RNASE1*, and 5, *NUCB2, RPLP0*, and *ABCG2*) in milk-purified mammary epithelial cells (MEC) **(B)** before (day –6) and during the first 4 days of differential milking frequency treatment. (A)** Data were expressed as mean daily milk yield per udder half. **(B)** Data were expressed as a % of variation during once-daily milking (ODM) in comparison with twice-daily milking (TDM). Six Holstein cows were subjected to unilateral ODM and TDM for 8 days. Before (day –6), and on the first 4 days of differential milking, 1.4 kg of milk was collected from both udder halves to prepare MEC by centrifugation and a specific purification process using an anti-cytokeratin antibody (K8.13) bound to magnetic beads. RNA from milk-purified MEC (150 ng) were reverse transcripted. Gene expressions were analyzed by real time PCR (Boutinaud et al., 2013a). For gene expression data, statistical analysis was performed on the semi-absolute mRNA molecule number of the target gene/PPIA reference gene ratio multiplied by 10^4^ and Log10 transformed. Milk yield and gene expression data were analyzed by ANOVA using the proc MIXED SAS procedure (SAS Institute Inc., Cary, NC) with REPEATED statements. Days were used as a repeated effect and cow (milking frequency) as the subject. The data obtained during the pretreatment period (day –6) for TDM were used as a covariate per udder half. The effects of milking frequency, different animals, number of days and the interaction between days, and milking frequency were tested. ^∗∗^*P* < 0.01, ^∗^*P* < 0.05, and ^τ^*P* < 0.10, symbols below a group of bars in bold correspond to ODM effect and symbols below each bar correspond to ODM effect at one day. Unpublished data.

The effect of ODM on mammary transcripts using milk MEC has also been studied in a short term kinetic experiment. Daily collection of milk during the first 4 days of unilateral ODM in six Holstein dairy cows made it possible to assess the pattern of expression for several genes, previously shown to be down-regulated during unilateral ODM in milk-purified MEC after 8 days of treatment (Boutinaud et al., 2013a). Milk yield was reduced from D1 (–20%) and this decrease stabilized at –30% from D2 to D4 (**Figure 2A**). Taking into account the unilateral milking effect for the 4 days of treatment, ODM induced down-regulation of gene expression for five transcripts (*RNASE1*, *SLC34A2*, *NUCB2, RPLP*0, and *ABCG2*) and a tendency for down-regulation for *LALBA* and *RNASE5* (**Figure 2B**). While milk yield was reduced from the first day, the effect on gene expression in milk-purified MEC was observed from the second day on three transcripts (*NUCB2, RPLP0*, and *ABCG2*), and these effects were still strong on the third day of differential milking. For three transcripts (*RNASE1* and *5* and *SLC34A2*), a tendency for down-regulation was observed on the third day of differential milking. In this study, one of the earliest and the strongest effect was observed for *ABCG2*, suggesting a pivotal role of *ABCG2* for controlling milk secretion during ODM. This effect is in agreement with the rapid down-regulation of *ABCG2* during mammary involution (Farke et al., 2008; Wu et al., 2014).

### The Effects of the Lactation Stage

The changes in transcripts according to the stage of lactation have been studied using milk-purified MEC in several species. Firstly, milk was collected from 24 multiparous Holstein–Friesian cows during 20 weeks of lactation to analyze the gene expression pattern of the six major milk protein mRNA (*CSN1S1, CSN1S2, CSN2*, *CSN3*, *LALBA*, and *LGB*, Sigl et al., 2012). Their expression was shown to peak during the first 2 weeks of lactation and decreased as lactation progressed. Accordingly, *LALBA* mRNA in milk-purified MEC from Holstein dairy cows was shown to decrease with the advanced stages of lactation (–80% between 21 and 52 weeks of lactation, Boutinaud et al., 2013b). The decrease in *LALBA* mRNA levels was associated with a 57% reduction in milk yield and a 15% reduction in lactose content (Boutinaud et al., 2013b). As during feed restriction, the decrease in the expression of major milk protein transcripts throughout the advanced stages of lactation was associated with the decrease of a key regulator of milk protein biosynthesis, namely ELF5 (Sigl et al., 2014).

In contrast to milk protein transcripts, *BAX* and *BCL2* transcripts were shown to rise as lactation progressed in milk-purified MEC of Holstein dairy cows (Boutinaud et al., 2013b). Accordingly in buffalo, an abrupt rise in *BAX* and *BCL2* transcripts was observed in milk-purified MEC during late lactation (Yadav et al., 2014). The effect of lactation stage has also been studied on the expression profiles of the lipogenic genes using milk-purified MEC in buffalo, which is one of the species among dairy animals with the richest fat content (Yadav et al., 2015). Interestingly, the expression of most of these genes was increased as the lactation progressed with the highest level at peak lactation and declined thereafter (3-Hydroxybutyrate dehydrogenase, Lipin1, Acetyl-coA synthetase short-chain family member 2), showing significant positive correlation with milk yield and a negative one with fat yield. In contrast, Acetyl-coA carboxylase alpha showed high expression during early lactation and a negative correlation with milk yield and a positive one with fat yield. Thus, genes involved in milk fat synthesis might be important regulators of milk and fat yields in buffalo.

### The Effects of Prolactin-release Inhibitor Treatment

Milk-purified MEC have been used to study the regulation of mammary transcripts in cows treated with an inhibitor of prolactin-release. Quinagolide is a dopamine agonist that inhibits the release of prolactin at milking (Lacasse et al., 2012). Quinagolide has been shown to induce a decrease in milk yield when injected to dairy cows (Lacasse et al., 2012). The effects of a Quinagolide treatment on mammary transcripts in milk-purified MEC have been studied in two cow experiments, one long term (9 weeks of treatment) and one short-term (5 days of treatment). In the long term experiment, the Holstein dairy cows were separated into two groups, one group (*n* = 5) received a daily injection of 1 mg Quinagolide and the control group (*n* = 4) received a daily injection of water for 9 weeks. Quinagolide administering resulted in a reduction in milk (–11.6%), lactose (–15.3%) and protein (–17.6%) yields. These decreases were associated with decreases in *LALBA* (–88%) and *CSN3* (–89%) gene expression in milk-purified MEC (Boutinaud et al., 2012). Quinagolide injections also induced a lower expression of long isoform prolactin receptor gene than in the control cows, suggesting a reduction in PRL sensitivity. In the short term experiment, nine Holstein dairy cows were assigned randomly to treatments during three periods of 5 days: (1) twice-daily, i.m. injections of 1 mg of Quinagolide; (2) injections of Quinagolide + twice-daily, i.v. injections of bovine recombinant prolactin at 2 μg/kg BW; (3) twice-daily injections of the vehicles as controls. Similarly to long term treatment, Quinagolide administering resulted in similar reductions in milk yields and in *LALBA* (–70%) and *CSN3* (–63%) mRNA levels. When prolactin was injected into Quinagolide treated cows, the mRNA levels of these genes were intermediate in comparison with those in control animals or Quinagolide treated cows, supporting the thought that prolactin positively promotes milk yield by regulating the expression of genes encoding milk proteins.

### The Effects of the Photoperiod

An experiment was carried out to identify the role undertaken by the regulation of genes for the control of milk calcium content under the influence of various photoperiods. It has recently been shown that milk calcium content decreases with long days (Boudon et al., 2013). Many proteins expressed in MEC are involved in the secretion of calcium into milk. Some of them are involved in calcium transport across cell membranes and others are known to bind to calcium or to play a role in the compartmentalization of calcium into Golgi or Endoplasmic Reticulum. The expression of several of these proteins has been studied using eight Holstein dairy cows that received 2-day length treatments (8 h of light/day for short days and 16 h/day for long days) in a Latin square design. After 11 days of light treatment provided by solarium lights (UVA and UVB), milk MEC were isolated by purification using anti-cytokeratin 1, 5, 10, and 14 antibodies bound to magnetic beads. The analysis of the milk’s composition showed that total and colloidal calcium contents in milk were lower during long days than during short days (*P* < 0.05). The gene expression analysis in milk-purified MEC showed that lower milk calcium content was associated with lower mRNA levels for *PMCA1*, a calcium transporter involved in transporting calcium across cell membranes (*P* < 0.05, Boutinaud et al., 2014). Concomitantly, the expression of two genes involved in calcium compartmentalization into Golgi (Secretory Pathway Ca^2+^ ATPase, *SPCA1*, and Inositol1,4,5, triphosphate receptor, *ITPR1*) were down-regulated during long days compared with short days (*P* < 0.05). The lower expression of these three calcium transporters in MEC could be responsible for lowering the milk calcium content during long days.

### The Relevance of Using the Milk-isolated MEC Method

When mammary transcript variations resulting from different factor (ODM, feed restriction, advancing lactation) were analyze using the milk-isolated MEC method, similar effects were found between studies. Concerning feed restriction, a consistent reduction in *SLC2A1* mRNA was observed in two studies in cows. Likewise, consistent reductions in *LALBA* mRNA levels were observed during ODM in several studies in both cows and goats. In addition, the effect of the stage of lactation had consistently reduced *LALBA* RNA levels, while increasing *BAX* RNA levels in cows and buffalo. Lastly, the reduction in *LALBA* and *CSN3* after Quinagolide injection was observed in two studies.

The relevance of using milk-isolated MEC method has been demonstrated thanks to the transcript variations, which were consistent with milk yield and composition. In most of the studies, the decrease in milk, lactose, protein, and casein yields were associated with a reduction in *LALBA* and *CSN3* gene expression in milk-purified MEC. This is the case during ODM in both bovine (Boutinaud et al., 2012, 2013a) and caprine species (Ben Chedly et al., 2011, 2013), during Quinagolide treatment (Boutinaud et al., 2012; Lollivier et al., 2015) and with advanced stages of lactation (Boutinaud et al., 2013b). The transcript variations in milk-purified MEC were also consistent with milk composition variations, as suggested, with the effect of photoperiod on the calcium transporters.

The opportunity of using milk MEC isolation allows repetitive sampling without damaging the mammary tissue. It is possible to determine gene expression profiles in the very same animal over the course of an experiment or a lactation period. Thus, milk MEC collection has been shown to enable kinetic studies. For example, the early effect of ODM could be investigated during the first 4 days of ODM application by daily collection of milk samples. Thus, we highlight a pivotal role of *ABCG2* during the first days of ODM application. Similarly, the pattern of some gene expression has been studied over the course of one lactation period in the same animal.

The purification of MEC from total milk cells has the advantage of making it possible to obtain epithelial cells only. In contrast to milk-isolated MEC, mammary tissue contains other types of cells (myoepithelial cells, endothelial cells, adipocytes, and fibroblasts). Despite the fact that mammary tissue essentially contains MEC, after RNA extraction, the presence of RNA from other types of cells may dilute the mammary transcripts of interest. For example, in the study where cows were injected daily with Quinagolide for 9 weeks, a reduction in milk protein mRNA was observed in mammary tissue after 4 weeks of treatment and was no longer significant after 8 weeks of treatment. After 9 weeks of treatment, the effect of Quinagolide on milk production was still present. Conversely to what was observed in mammary tissue after 8 weeks, lower levels of *LALBA* and *CSN3* mRNA were still observed in the milk-isolated MEC from the Quinagolide-treated cows in comparison with the controls after 9 weeks of treatment. Analyzing mammary transcripts in milk-purified MEC would be a more sensitive method for detecting low gene expression changes, as the purification step makes it possible to obtain MEC only. Results regarding the effect of ODM suggest the same idea. The effect of ODM on *LALBA* gene expression in cows is less significant in mammary biopsies than in milk-purified MEC (**Table 1**). Similarly, the percentage of *LALBA* gene expression variation in mammary biopsy is lower than in milk-purified MEC in two studies in goats (**Table 2**). MEC in milk may be more representative of a late secretory status, and may represent cells that are in full secretion whereas MEC of different status are present in biopsies. However, overall higher changes in gene expression with milk-purified MEC compared than with mammary tissue, might be due to a more pronounced senescence, MEC being shed into milk when they become senescent.

One of the limitations of using milk-isolated MEC to analyze mammary gene expression is that a big volume of milk is needed (1.8–3.6 kg of bovine milk) and it limits the number of samples processed at one time. Moreover the fact that the purification of MEC from milk has to be done on fresh milk is another limitation of the methodology. Even though the proteome profile of milk-purified MEC has been recently studied in zebu (Janjanam et al., 2013), the amount of MEC collected limit the number of different types of analysis that can been done on the same sample. In contrast, mammary biopsy allows performing, in addition to RNA analysis, protein, and immunohistochemical analyses. Moreover milk production also depends on extra-epithelial elements such as the vascular function and angiogenesis that can’t be considered using a method with which only MEC are collected. In addition to these technical limitations, the lack of knowledge about milk-isolated MEC identity may limit the use of this method in replacement of other types of mammary cell collection method. Indeed, even if the majority of milk isolated cells are alive, some others are apoptotic MEC shed into milk due to a turnover of the secretory tissue (Herve et al., 2015). Besides that, milk should contain both ductal and secretory MEC, the content in secretory MEC being probably higher than for ductal MEC as a consequence of physical pressure in the alveolus associated with the continued filling and emptying cycle associated with milk synthesis and myoepithelial contraction during milking. This is supported by the proteome analysis of MEC isolated from zebu milk that showed the expression profile of proteins involved in lactation process suggesting that most these cells are metabolically active and secretory cells (Janjanam et al., 2013). From our knowledge, until now, no cell culture was performed after MEC immuno-purification from milk. In contrast MEC was cultivated directly after somatic cells isolation from milk by centrifugation (Ben Chedly et al., 2010; Sorg et al., 2012). Additional studies via culture of milk-purified MEC could contribute to better characterize these cells, on for example the phosphorylation status and on protein levels as suggested by Sigl et al. (2012) and as performed in human after mammary biopsy (Gudjonsson et al., 2002). Few is known about the epigenetical status of milk-isolated MEC, except that their global DNA methylation was found quite variable between samples (on average 67 ± 8%) and lower than peripheral blood mononuclear cell (Gasselin et al., 2015). Thus a better characterization of the different types of MEC present in milk has yet to be investigated further.

## Conclusion

The isolation of MEC from milk has been shown to have several purposes. It is a means of assessing regulation of transcription by kinetic studies. One of the interests of using milk-purified MEC compared with using total milk somatic cells is the possibility to study genes not specifically expressed in MEC. Moreover, the use of milk MEC purified by immunomagnetic separation may also be more accurate to analyze mammary transcript levels than mammary biopsy. The fact that consistent transcript variations with milk yield and composition variations were observed, and that consistent effects of milking frequency, feed restriction, stage of lactation, and Quinagolide treatment shows evidence of the relevance and the efficiency of using purified MEC for ruminants. However, RNA from milk-isolated MEC is sensitive to degradation. This could explain the differences obtained between milk-isolated MEC and mammary biopsy in two studies. As a conclusion, when RNA quality is conserved, MEC isolated from milk are a valuable, non-invasive source of mammary mRNA which can be used to study various factors of milk yield and composition variations (ODM, feeding level, endocrine status, photoperiod modulation, and stage of lactation). Although other promising methods of recovering and analyzing mammary transcripts such as using milk fat globules have been developed (Maningat et al., 2007, 2009; Brenaut et al., 2012), the use of purified MEC is still advantageous as it can determine the level of exfoliation of MEC from the mammary tissue, indicating the regulation of mammary cell number and the long term effect on milk yield as recently reviewed (Herve et al., 2015). Finally, such a method of cell isolation would provide a promising opportunity to further understand the involvement of microRNA, small non-coding RNA, in the lactation process (Mobuchon et al., 2015; Wicik et al., 2015) and, for example, their role in the regulation of mammary host defense (Lawless et al., 2013; Jin et al., 2014).

## Author Contributions

Contributions to the conception of the review: MB, LH, and VL.

Acquisition, analysis, and interpretation of unpublished data for the review: MB.

Drafting the work: MB, LH, and VL.

## Conflict of Interest Statement

The authors declare that the research was conducted in the absence of any commercial or financial relationships that could be construed as a potential conflict of interest.
